# Connexin-dependent gap junction enhancement is involved in the synergistic effect of sorafenib and all-*trans* retinoic acid on HCC growth inhibition

**DOI:** 10.3892/or.2013.2894

**Published:** 2013-12-05

**Authors:** YAN YANG, SHU-KUI QIN, QIONG WU, ZI-SHU WANG, RONG-SHENG ZHENG, XU-HUI TONG, HAO LIU, LIANG TAO, XIAN-DI HE

**Affiliations:** 1 Department of Medical Oncology, The First Affiliated Hospital of Bengbu Medical College, Bengbu 233004, P.R. China; 2 Department of Intensive Care, The First Affiliated Hospital of Bengbu Medical College, Bengbu 233004, P.R. China; 3 Department of Medical Oncology, PLA Cancer Center, Nanjing Bayi Hospital, Nanjing 210002, P.R. China; 4 Department of Pharmacy, Bengbu Medical College, Bengbu 233000, P.R. China; 5 Department of Pharmacology, Zhongshan School of Medicine, Sun Yat-Sen University, Guangzhou 510080, P.R. China

**Keywords:** sorafenib, all-*trans* retinoic acid, growth inhibition, gap junction, hepatocellular carcinoma

## Abstract

Increasing gap junction activity in tumor cells provides a target by which to enhance antineoplastic therapies. Previously, several naturally occurring agents, including all-*trans* retinoic acid (ATRA) have been demonstrated to increase gap junctional intercellular communication (GJIC) in a number of types of cancer cells. In the present study, we investigated *in vitro* whether ATRA modulates the response of human hepatocellular carcinoma (HCC) cells to sorafenib, the only proven oral drug for advanced HCC, and the underlying mechanisms. HepG_2_ and SMMC-7721 cells were treated with sorafenib and/or ATRA, and cell proliferation and apoptosis were analyzed; the role of GJIC was also explored. We found that ATRA, at non-toxic concentrations, enhanced sorafenib-induced growth inhibition in both HCC cell lines, and this effect was abolished by two GJIC inhibitors, 18-α-GA and oleamide. Whereas lower concentrations of sorafenib (5 μM) or ATRA (0.1 or 10 μM) alone modestly induced GJIC activity, the combination of sorafenib plus ATRA resulted in a strong enhancement of GJIC. However, the action paradigm differed in the HepG_2_ and SMMC-7721 cells, with the dominant effect of GJIC dependent on the cell-specific connexin increase in protein amounts and relocalization. RT-PCR assay further revealed a transcriptional modification of the key structural connexin in the two cell lines. Thus, a connexin-dependent gap junction enhancement may play a central role in ATRA plus sorafenib synergy in inhibiting HCC cell growth. Since both agents are available for human use, the combination treatment represents a future profitable strategy for the treatment of advanced HCC.

## Introduction

Hepatocellular carcinoma (HCC) is the sixth most common solid tumor in humans worldwide and the third most common cause of cancer-related death (1). Chemotherapy is unsafe for many HCC patients, since most HCCs develop on the basis of cirrhosis, and chronically damaged or cirrhotic underlying livers poorly tolerate conventional chemotherapy. There is thus the need for non-toxic, novel therapies for HCC patients. Sorafenib is a multi-kinase inhibitor with high efficacy against a variety of cancers as confirmed in preclinical models (2). It suppresses cell proliferation and induces apoptosis in HCC cell lines (3,4) and has become the first approved drug for advanced HCC by the positive results of clinical trials (5,6). Sorafenib has been reported to inhibit experimental HCC cell growth and angiogenesis by inhibiting Raf kinase as well as receptor tyrosine kinases, such as VEGF and PDGF receptors (3,7). Unlike traditional systemic chemotherapy, sorafenib has shown survival benefits but only minimal tumor shrinkage (8). Moreover, substantial severe, although rare, adverse events such as cardiac ischemia, hand-foot syndrome, neutropenia and hypertension are associated with the use of this drug. Thus, there is increasing interest in manipulating its actions to reduce its toxicity, as well as in examining the effects of this drug in combination with other agents (9).

All-*trans* retinoic acid, an analog of vitamin A, is currently being extensively studied for its potential as a therapeutic and chemopreventive agent since it has been used successfully in the treatment of acute promyelocytic leukemia (APL) (10) and other hematologic diseases (11,12). It induces cellular differentiation of numerous malignant tumors and inhibits their growth (13). Notably, epidemiological evidence indicates that low levels of serum retinol are correlated with HCC risk (14,15), suggesting a potential role of retinoids in the chemoprevention of this cancer. Consistent with this view, *in vitro* and *in vivo* preclinical evidence indicated that ATRA was able to inhibit proliferation and induce apoptosis in human hepatoma cells (Hep3B) (16) and suppressed tumorigenicity in a nude mouse model (17). The antiproliferative, differentiation and/or apoptotic effects of ATRA on rat hepatocytes (18) or HepG_2_ cells (19,20), have also been demonstrated. The precise mechanism through which ATRA exerts its chemopreventive effect remains controversial. Several studies have indicated that the antitumor effects of ATRA in various types of cancers are associated with its ability to restore gap junction function of otherwise gap junctional communication-impaired tumor cells (21,22).

Gap junctions, composed of connexins (Cxs), connect the cytoplasm of neighboring cells, thereby mediating the direct exchange of cytoplasmic signaling molecules smaller than 1 kDa, such as secondary message Ca^2+^, cyclic adenosine monophosphate (cAMP) and inositol triphosphate (IP_3_) between adjacent cells. This process of exchange of molecules between neighboring cells via gap junctions is termed gap junctional intercellular communication (GJIC). GJIC has been implicated in many cellular functions, including cancer biology and chemotherapy (23,24). Emerging evidence indicates a GJIC-dependent enhancing effect on the toxicity of various chemotherapeutic agents in tumor cells (25–27). In this context, an intercellular diffusion of toxic/apoptotic signals through gap junction channels is considered to be involved. The degree of the additive bystander toxicity generally correlates with the level of GJIC, although it is present at low levels or even completely absent in tumor cells (28,29). Therefore, increasing gap junction activity in tumor cells provides a new target by which to enhance antineoplastic therapies.

In view of these observations, there has been considerable interest in using the combination of sorafenib and ATRA for the prevention and treatment of HCC. The aim of this study was to investigate whether the combination of sorafenib plus ATRA exerts more pronounced growth-inhibitory effects on human HCC cells and to examine possible mechanisms for such an effect, predominantly focusing on the modulation of GJIC by the combination of these two agents.

## Materials and methods

### Materials

Sorafenib was obtained from the Bayer Corp. (West Haven, CT, USA). All-*trans* retinoic acid (ATRA), 18-α-glycyrrhetinic acid (18-α-GA), oleamide, dimethyl sulfoxide (DMSO), 3-(4,5-dimethylthiazol-2-yl)-2,5-diphenyltetrazolium bromide (MTT), anti-Cx32 and anti-Cx43 mouse IgG were from Sigma (St. Louis, MO, USA). Dulbecco’s modified Eagle’s medium (DMEM), fetal bovine serum, TRIzol, cell labeling dyes CM-DiI and calcein-AM (acetoxymethyl ester), anti-Cx26 mouse IgG and fluorescein isothiocyanate (FITC)-anti-mouse IgG were from Invitrogen (Carlsbad, CA, USA). Secondary antibodies for western blotting were from Amersham Biosciences Corp. (Piscataway, NJ, USA). All other reagents were from Sigma unless stated otherwise.

### Cell lines and cell culture

Human HCC cell line HepG_2_ was obtained from the American Type Culture Collection (Manassas, VA, USA), and SMMC-7721 was purchased from the cell bank of the Shanghai Institutes for Biological Sciences. Both cell lines were grown at 37°C in a humidified atmosphere containing 5% (v/v) CO_2_ in DMEM supplemented with 10% fetal bovine serum, and 100 U/ml streptomycin and 100 mg/ml penicillin.

### Drug treatment

Sorafenib and ATRA were dissolved in DMSO to a stock concentration of 10 and 50 mM, respectively. A stock concentration of 18-α-GA (10 mM) or oleamide (25 mM) was also diluted in DMSO and stored at −20°C. Just before each experiment, aliquots were thawed and diluted to the desirable concentration with DMEM. The final concentration of DMSO as solvent was always <0.1%.

### MTT assay

HepG_2_ (2×10^4^ cells/well) and SMMC-7721 (8×10^3^ cells/well) cells were seeded into 96-well plates for 1 day, and then exposed to sorafenib and ATRA either alone or in combination at the indicated concentrations for 48 h. Cells incubated with DMSO at the same concentration (always less than 0.1% v/v) were used as a control. MTT (5 mg/ml in PBS) was then added to each well, and the dishes were incubated at 37°C for 4 h, and the medium containing MTT was then removed. The formazan crystals in the viable cells were solubilized with 100 μl DMSO, and the absorbance at 490 nm of each well was read using a microplate enzyme-linked immunosorbent assay (ELISA) reader (MRX II; Dynex Technologies, Chantilly, VA, USA). All experiments were performed at least three times, with five wells for each concentration of the tested compounds (n=5 per experiment). The cell viability was calculated as follows: (OD of experimental group - OD of blank group) / (OD of control group - OD of blank group).

### Hoechst 33258 staining

The staining method was carried out according to the method recommended by the manufacturer. In brief, after treatment for 48 h, HepG_2_ and SMMC-7721 cells cultured in 6-well plates were washed with PBS and fixed with 4% paraformaldehyde for 30 min at room temperature. Fixed cells were washed with PBS, stained with Hoechst 33258 (Sigma, 5 μg/ml) for 30 min at room temperature in the dark, and the apoptotic cells were identified by condensation and fragmentation of nuclei as examined by a fluorescence microscope (Olympus, Tokyo, Japan). The apoptotic rate of the cell population was calculated as the ratio of apoptotic cells to total cells counted ×100. A minimum of 500 cells were counted for each treatment.

### Early stage of cell apoptosis by flow cytometric analysis

Early stage of cell apoptosis was measured by Annexin V-FITC and propidium iodide (PI) (BD Biosciences Clontech, USA) labeling technique and flow cytometric analyses. Cells were plated onto 12-well plates and grown to confluence. They were then treated in the absence (vehicle control) or presence of sorafenib (10 μM), ATRA (10 μM for HepG2 cells and 0.1 μM for SMMC-7721 cells), or sorafenib + ATRA for 8 h. Cells were harvested, washed with cold PBS and then resuspended in 100 μl binding buffer containing 5 μl Annexin V-FITC and 10 μl of PI. After an incubation time of 10 min at room temperature in the dark, stained cells were analyzed by flow cytometry. Unstained and single stained controls were included in each experiment. This assay was carried out in triplicate.

### Determination of GJIC: ‘parachute’ dye-coupling assay

GJIC was determined by a ‘parachute’ technique as we previously described (27,30). Briefly, cells were cultured in 12-well plates to 80–85% confluence. Donor cells from one well were incubated with a freshly made solution of 5 μM calcein-AM and 2.5 μM CM-DiI in growth medium for 30 min at 37°C. CM-DiI is a nontransferable membrane dye that does not spread to coupled cells, while calcein-AM is converted intracellularly into the gap junction-permeable dye calcein. Unincorporated dye was removed by three consecutive washes with culture medium. The donor cells were then trypsinized and seeded onto the receiver cells at a 1:150 donor/receiver ratio. The donor cells were allowed to attach to the monolayer of receiver cells and form gap junctions for 4 h at 37°C, and then examined with a fluorescence microscope (Olympus). The average number of receiver cells containing calcein per donor cell was considered as a measure of the degree of GJIC.

### Western blotting

Cells were washed three times with cold PBS and lysed in lysis buffer (Tris-HCl pH 7.4 20 mM, NaCl 150 mM, EDTA 1 mM, EGTA 1 mM, Triton 1%, sodium pyrophosphate 2.5 mM, Na_3_VO_4_ 1 mM, β-glycerophosphate 1 mM, protease inhibitors 1:1,000), followed by a brief sonication. The suspension was then centrifuged at 12,000 rpm for 30 min at 4°C, and proteins from the supernatant were extracted. Protein determination was performed using a DC protein assay kit (Bio-Rad Chemical Co.). Samples (25 μg) from cells were applied to SDS-polyacrylamide gels of 10% (w/v) acrylamide, followed by electrophoresis and blotting. Membranes were blocked in 5% nonfat milk for 30 min at room temperature and probed with appropriate antibodies at the dilution recommended by the suppliers. The immunoreactive bands were visualized using an enhanced chemiluminescence detection kit (Amersham, Aylesbury, UK).

### Analysis of immunofluorescence

Cells were seeded onto sterile slides with coverslips in 24-well plates and the indicated treatments were carried out. The cells were then briefly washed three times with PBS, and fixed with 0.1% Triton X-100–4% paraformaldehyde for 30 min. Coverslips were blocked with 2% bovine serum albumin (BSA) in PBS and probed with anti-Cx32 (1:100) or anti-Cx43 (1:200), the primary antibodies diluted in 2% BSA in PBS overnight at 4°C. Cells were washed, followed by the addition of FITC anti-mouse IgG at a 1:200 dilution in 2% BSA in PBS for 2 h in the dark at room temperature. Nuclear staining was performed with DAPI at 37°C for 5 min. After rinsing, the coverslips were mounted on slides, and the cells were examined under a fluorescence microscope (Olympus). Six to eight randomly selected fields were examined in each of three separate experiments.

### RNA isolation and reverse transcriptase-polymerase chain reaction (RT-PCR)

Total RNA was extracted using Trizol reagent according to the manufacturer’s instructions. Complementary DNA (cDNA) was synthesized from 1 μg RNA using the standard procedure with avian myeloblastosis virus reverse transcriptase (Promega) to generate 20 μl of cDNA at 42°C for 60 min. For polymerase chain reaction (PCR) quantification, 2 μl of cDNA reaction was amplified in a 20 μl standard PCR reaction. PCR was initiated at 94°C for 3 min followed by 30 cycles consisting of 45 sec at 94°C, 45 sec at 58°C, and 45 sec at 72°C, with the final cycle extended to 10 min at 72°C, followed by termination at 4°C. The following primers were used: for human Cx32, forward primer 5′-TCC CTGCAGCTCATCCTAGT-3′ and reverse primer 5′-CCC TGAGATGTGGACCTTGT-3′, product size 156 bp; for human Cx43, forward primer 5′-AGGAGTTCAATCACTTGGCG-3′ and reverse primer 5′-GCAGGATTCGGAAAATGAAA-3′, product size 168 bp; for human β-actin, forward primer 5′-TCCTCCTGAGCGCAAGTACTC-3′ and reverse primer 5′-GCATTTGCGGTGGACGAT-3′, product size 130 bp. The detection of β-actin transcripts provided an internal control in PCR, standardizing the quantity of input cDNA. PCR products were analyzed on an ethidium bromide-stained 1.5% agarose gel.

### Statistical analysis

The data are represented as means ± SEM for the number of individual experiment specified in each figure legend. All statistical analyses used SigmaPlot 10.0 software (Jandel Scientific, San Rafael, CA, USA). Comparison of numerical data was achieved with the unpaired Student’s t-test; differences with p<0.05 were considered significant.

## Results

### Inhibition of HCC cell growth by sorafenib plus ATRA

**Figure 1:**
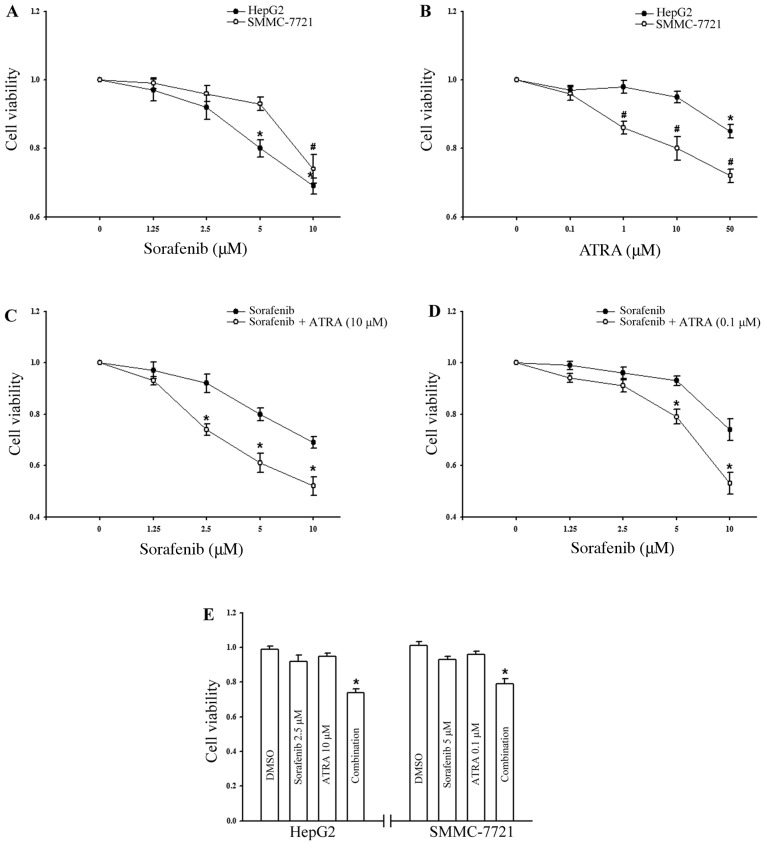
Effects of sorafenib and ATRA on the growth inhibition in HCC cell lines. Growth inhibition of (A) HepG_2_ and (B) SMMC-7721 cells following treatment with a range of sorafenib or ATRA concentrations for 48 h. Growth inhibition of (C) HepG_2_ and (D) SMMC-7721 cells following treatment with the combination of ATRA at its most nontoxic concentration, and varying concentrations of sorafenib. (E) Growth inhibition of HepG_2_ and SMMC-7721 cells by sorafenib, ATRA or the combination, both at their most nontoxic concentrations. Data represent the mean ± SEM of four independent experiments. ^*^p<0.05 or ^#^p<0.05 vs. vehicle control (A and B); ^*^p<0.05 vs. sorafenib treatment alone (C and D); ^*^p<0.05 vs. single-agent treatment (E).

To assess the growth-inhibitory effects of combining sorafenib with ATRA, HepG_2_ human HCC cells, which are frequently used in evaluations of the effect of sorafenib, were treated with the tested agents, either individually or in combination, and were then examined by MTT assay. The results shown in Fig. 1A and B demonstrated that neither sorafenib nor ATRA alone had a significant effect on HepG_2_ cell viability at the tested concentrations (sorafenib up to 2.5 μM; ATRA up to 10 μM). We then focused on the effect of ATRA at a non-toxic concentration on sorafenib-induced cell inhibition. Fig. 1C shows the data for the combination of varying doses of sorafenib and a fixed dose of ATRA, demonstrating that the growth inhibitory activity of each treatment was enhanced by the addition of ATRA (p<0.05). Similar results were also observed for the human HCC cell line SMMC-7721 of Asian origin (Fig. 1D); the response to sorafenib was less sensitive while the response to ATRA was markedly sensitive (Fig. 1A and B). It is noteworthy that in both cell lines, the combination of these two agents both at non-toxic concentrations significantly inhibited cell growth when compared to the effect of each single agent treatment (p<0.05, Fig. 1E).

### Induction of apoptosis by sorafenib plus ATRA

**Figure 2:**
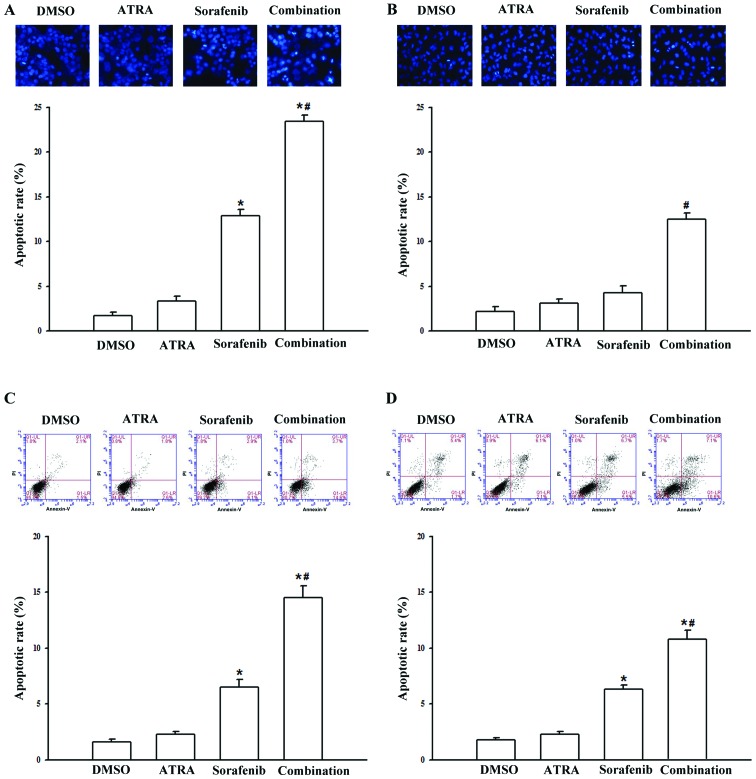
Induction of cell apoptosis by the combination of sorafenib and ATRA. (A) HepG_2_ and (B) SMMC-7721 cells were treated with sorafenib (5 μM), ATRA (10 μM for HepG_2_ cells and 0.1 μM for SMMC-7721 cells) alone or in combination for 48 h, after which the late stage of cell apoptosis was assessed by Hoechst 33258 staining assay. (C) HepG_2_ and (D) SMMC-7721 cells were treated with sorafenib (10 μM), ATRA (10 μM for HepG_2_ cells and 0.1 μM for SMMC-7721 cells) alone or in combination for 8 h, after which the early stage of cell apoptosis was measured by flow cytometric analysis. Data represent the mean ± SEM of three independent experiments, and representative images are shown. The apoptosis of cells was observed at a magnification of ×20. ^*^p<0.05 vs. vehicle control; ^#^p<0.05 vs. single-agent treatment.

We next investigated the effect of the combination treatment of sorafenib plus ATRA on cell apoptosis. Morphological change showing the presence of late apoptosis was assessed by Hoechest 33258 staining. Data showed that treatment with sorafenib alone, at a low concentration (5 μM) for 48 h was sufficient to trigger slight apoptosis in the HepG_2_ cell line (12.9±0.6%), but led to no significant effect on SMMC-7721 cells. However, simultaneous exposure to sorafenib and ATRA, at its maximum non-toxic concentrations, respectively (10 μM for HepG2 cells and 0.1 μM for SMMC-7721 cells), resulted in a pronounced increase in apoptosis (Fig. 2A and B). Annexin V/PI double staining followed by flow cytometric analysis, which provides more sensitivity and precision, was conducted to detect the early stage of cell apoptosis. As shown in Fig. 2C and D, to yield a modest toxic effect, sorafenib, at a high concentration of 10 μM, was used. Compared with either agent alone, the apoptotic rate was substantially greater following the treatment of the cells with sorafenib and ATRA concomitantly for a short treatment time (8 h). These highly concordant results indicated that ATRA synergistically potentiated sorafenib to induce apoptosis in both cell lines.

### Involvement of GJIC in sorafenib plus ATRA-mediated apoptosis

**Figure 3:**
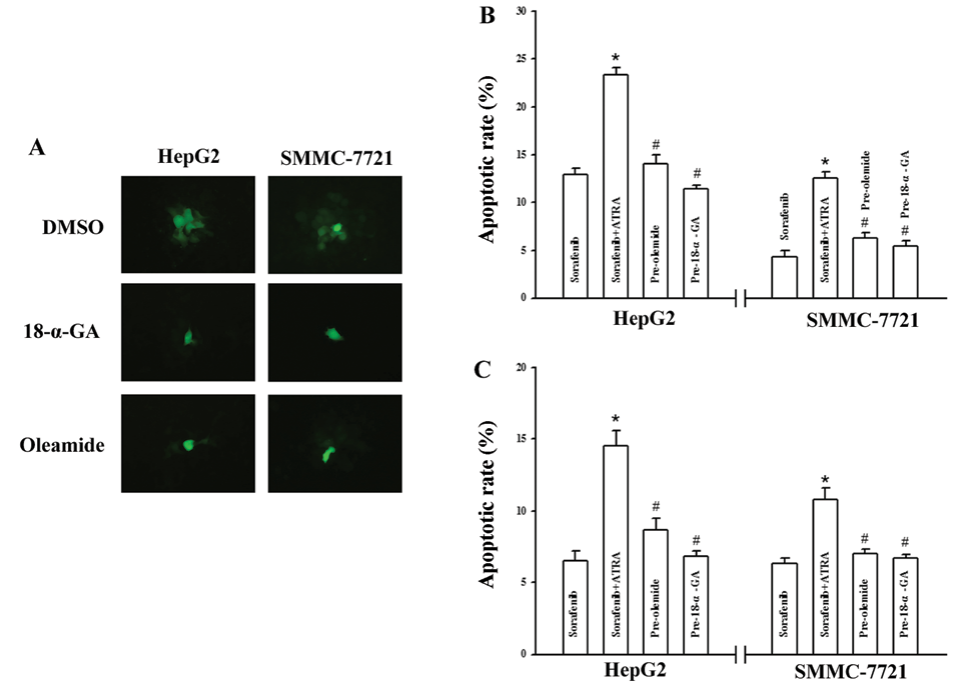
Involvement of GJIC in the induction of apoptosis by the combination of sorafenib and ATRA. (A) Fluorescence images show the degree of dye coupling by the parachute assay. 18-α-GA (10 μM) and oleamide (25 μM) decreased GJIC in both cell lines. Cells were observed at a magnification of ×40. (B) Cells were treated with sorafenib (5 μM), sorafenib plus ATRA (10 μM for HepG_2_ cells and 0.1 μM for SMMC-7721 cells) for 48 h, or pretreated with GJIC inhibitors for 1 h and then incubated with sorafenib plus ATRA for an additional 48 h, and cell apoptosis was subsequently determined using Hoechst 33258 staining. (C) Cells were treated with sorafenib (10 μM), sorafenib plus ATRA (10 μM for HepG_2_ cells and 0.1 μM for SMMC-7721 cells) for 8 h, or pretreated with GJIC inhibitors for 1 h and then incubated with sorafenib plus ATRA for an additional 8 h, and cell apoptosis was subsequently determined, using flow cytometric analysis. Data represent the mean ± SEM of three independent experiments. ^*^p<0.05 vs. sorafenib treatment alone; ^#^p<0.05 vs. combination treatment with sorafenib and ATRA.

GJIC plays a critical role in cancer therapy and previous findings suggest that ATRA augments gap junction function in a variety of normal and malignant cell types (21,22,31); therefore, we aimed to ascertain whether the mechanism involved in sorafenib plus ATRA-mediated apoptosis is related to alteration of GJIC. Although few drugs have been identified as highly potent and selective gap junction channel-blocking molecules (32), two extensively used drugs as GJIC blockers, 18-α-GA (33,34) and oleamide (35,36), serve as a powerful tool with which to investigate the role of GJIC in the process. As shown in Fig. 3A, HepG_2_ and SMMC-7721 cells were communication-competent and transferred calcein to numerous cells distant to the ‘donor cell’, and incubation with 18-α-GA (10 μM) or oleamide (25 μM) for 1 h, prevented GJIC in both cell types as assessed by the dye transfer assay. Moreover, pretreatment of the two HCC cell lines with either inhibitor completely reversed the induction of apoptosis caused by the combination of the agents as measured by Hoechst 33258 staining and flow cytometric analysis (Fig. 3B and C).

### Enhancement of GJIC by sorafenib plus ATRA

**Figure 4:**
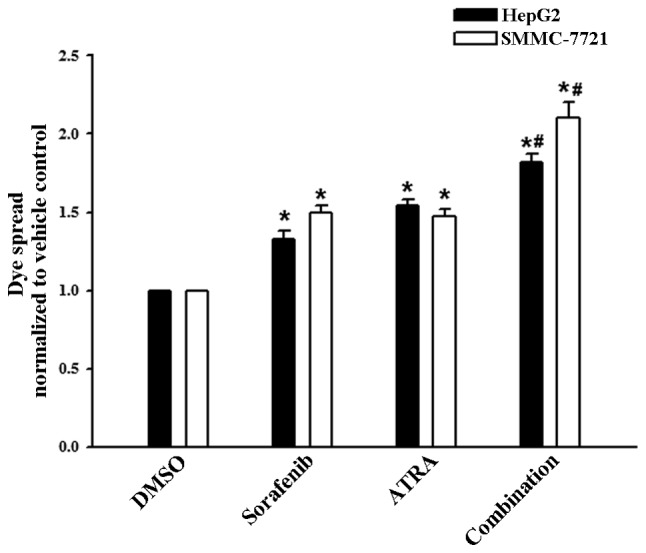
Effects of sorafenib and ATRA on GJIC activity in HCC cell lines. HepG_2_ and SMMC-7721 cells were treated with sorafenib (5 μM), ATRA (10 μM for HepG_2_ cells and 0.1 μM for SMMC-7721 cells), or in combination for 48 h. GJIC was assessed as the average number of receiver cells containing calcein from each donor cell, normalized to vehicle controls treated with DMSO. Data represent the mean ± SEM of four independent experiments. ^*^p<0.05 vs. vehicle control; ^#^p<0.05 vs. single-agent treatment.

The blockade of the sorafenib plus ATRA-induced apoptosis in HCC cells by GJIC inhibitors indicated that the enhanced growth inhibitory effect was mediated by GJIC. To test this hypothesis, the effects on GJIC by sorafenib plus ATRA or either agent alone were investigated. Results of the dye transfer assay are summarized in Fig. 4. Treatement with sorafenib (5 μM) as well as ATRA (10 μM) substantially resulted in up to a 33 and 54% increase in the dye spread between the HepG_2_ cells respectively, and the most pronounced effects were expectedly achieved by the combination treatment. A similar, yet more marked effect was observed when SMMC-7721 cells were treated with sorafenib (5 μM), ATRA (0.1 μM), or in combination for 48 h, with the rate of improvement being 50±4.1, 47±5.3 and 110±10.2%, respectively (Fig. 4).

### Effect on Cx expression by sorafenib plus ATRA

**Figure 5:**
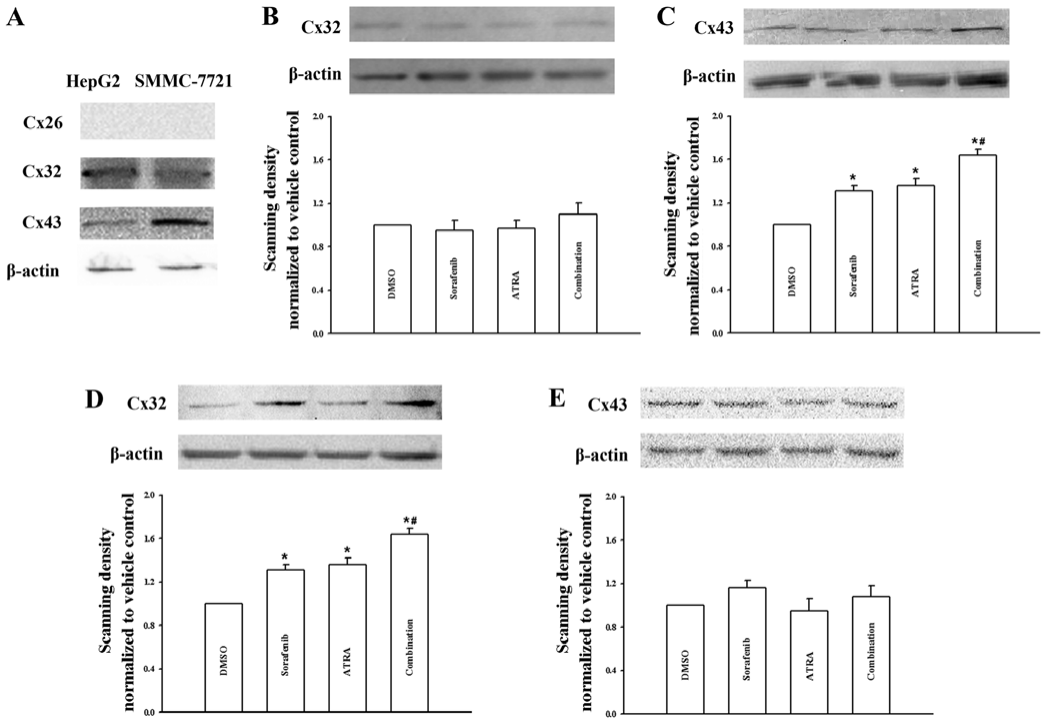
Effects of sorafenib and ATRA on the protein expression of Cxs. (A) Expression of different Cx isoforms in HepG_2_ and SMMC-7721 cells, as shown by western blotting for Cx26, Cx32 and Cx43 proteins, respectively. (B and C) Sorafenib (5 μM), ATRA (10 μM), or in combination increased the expression of Cx43, but not Cx32, after treatment for 48 h in HepG_2_ cells. (D and E) Sorafenib (5 μM), ATRA (0.1 μM), or in combination increased the expression of Cx32, but not Cx43, after treatment for 48 h in SMMC-7721 cells. Bar graphs were plotted according to the densitometry of the Cx/actin band densities, normalized to vehicle controls treated with DMSO. Data represent the mean ± SEM of three independent experiments. ^*^p<0.05 vs. vehicle control; ^#^p<0.05 vs. single-agent treatment.

Since the dye transfer assay measures the permeability of gap junction, the enhancement in GJIC activity could be a consequence of either gating state or the number of Cx channels on the cell membrane (28). We thus investigated the modulation of Cx expression in the two HCC cell lines. As shown in Fig. 5A, both cell types had no endogenous Cx26 expression virtually, whereas a moderate level of Cx32 or Cx43 protein was detected. The results were consistent with prior studies (31,37). We then focused on the expression modulation of these two structural proteins. In the HepG_2_ cell line, no Cx32 expression change was detected following each treatment, whereas treatment with sorafenib or ATRA alone elicited a slight stimulation of Cx43 expression. A significant increase in the Cx43 amount was achieved following treatment with the combination of sorafenib plus ATRA (Fig. 5B and C). By contrast, the enhancement of GJIC activity in SMMC-7721 cells was associated with an increase in Cx32 expression, but not Cx43 expression, and this effect was obvious following the combination treatment (Fig. 5D and E).

### Effect on Cx distribution by sorafenib plus ATRA

**Figure 6:**
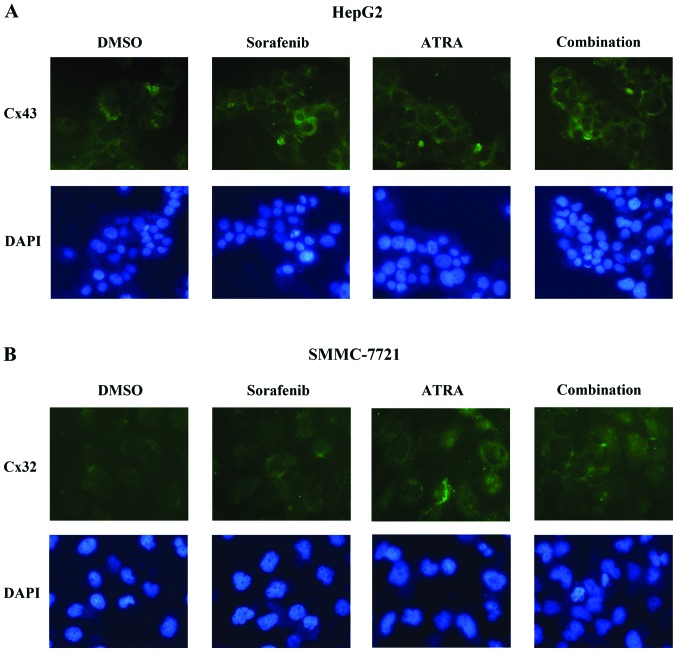
Immunofluorescence staining of (A) Cx43 in HepG_2_ cells and (B) Cx32 in SMMC-7721 cells following exposure to sorafenib (5 μM), ATRA (10 μM for HepG_2_ cells and 0.1 μM for SMMC-7721 cells), or in combination for 48 h. Improved localization of fluorescent spots is noted, presumably corresponding to gap junctions on the plasma membrane of treated cells, compared with those of the vehicle controls. Representative images from three independent experiments are shown. Cells were observed at a magnification of ×40.

Since increased intracellular levels of Cxs do not necessarily imply that these proteins are also properly assembled to form functional gap junction channels on the plasma membrane, we therefore evaluated Cx relocalization and positioning in both HCC cell lines. As shown in Fig. 6A, HepG_2_ cells displayed a small number of Cx43-specific positive spots, predominantly along the plasma membrane at cell-cell contacts, confirming the presence of functional GJIC in intact cells (Fig. 3A). Following the treatment of sorafenib or ATRA, Cx43 staining increased and was located throughout the cell membrane and cytoplasm. The most marked increment of Cx43 in both compartments was observed following the combination treatment for 48 h (Fig. 6A), consistent with the immunoblot data (Fig. 5C). In the SMMC-7721 cells, the treatment produced similar findings demonstrating an increase in the membrane-associated levels of Cx32 staining (Fig. 6B). These results suggest that the number of gap junctions composed of its structural Cx was paralleled by an increase in the amount of protein.

### Modulation of Cx32 and Cx43 mRNA expression

**Figure 7:**
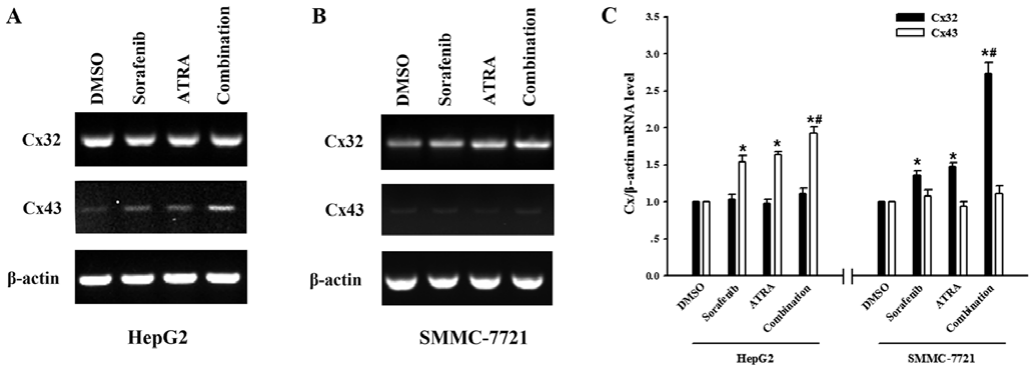
Effects of sorafenib and ATRA on the mRNA expression of Cxs. Agarose gel electrophoresis of RT-PCR products in (A) HepG_2_ and (B) SMMC-7721 cells following treatment with sorafenib (5 μM), ATRA (10 μM for HepG_2_ cells and 0.1 μM for SMMC-7721 cells), or in combination for 48 h. (C) Quantification of mRNA level indicated that the mRNA expression of Cx43, but not Cx32, was increased by each treatment in HepG_2_ cells, while the mRNA expression of Cx32, but not Cx43 was increased by each treatment in SMMC-7721 cells. Data represent the mean ± SEM of three independent experiments. ^*^p<0.05 vs. vehicle control; ^#^p<0.05 vs. single-agent treatment.

To further test whether the modulation of Cx expression occurred at the transcriptional level, we measured the expression of Cx32 and Cx43 mRNA in cells treated with sorafenib and ATRA, either alone or in combination. As shown in Fig. 7A and C, HepG_2_ cells displayed no appreciable change in the amplified PCR product for the Cx32 transcript in either sample, whereas enhancement of Cx43 mRNA expression to different degrees was detected. In regards to the SMMC-7721 cells, densitometric analysis demonstrated stable mRNA expression of Cx43 following each treatment, whereas the combination of sorafenib and ATRA caused a most significant augmentation in the Cx32 mRNA level, when compared with either agent alone (Fig. 7B and C). This trend was highly consistent with the effect as demonstrated for the drug-induced increases in Cx protein level (Fig. 5).

## Discussion

The present study investigated the effects of sorafenib in combination with ATRA on the inhibition of growth and induction of apoptosis in human HCC cells, and the involvement of gap junctional communication in facilitating this effect. The results showed that an upregulation of GJIC between treated cells was associated with the observed pronounced growth-inhibitory effects by ATRA in combination with low and clinically relevant concentrations of sorafenib. Thus, the combination treatment may be a candidate for clinical application, either to permit the use of lower and less toxic doses of sorafenib, or to supplement standard sorafenib doses in order to enhance clinical responses, that have, to date, been poor.

Although clinical trials (5,6) have established sorafenib as the first standard systemic therapy for advanced and unresectable HCC, the survival benefit of sorafenib is still unsatisfactory, and patients with cirrhotic livers exhibit poor tolerance to this drug. Consequently, the development of novel approaches that exhibit a non-toxic effect or have low toxicity when using sorafenib for the treatment of HCC, particularly the establishment of effective combination therapies, is required. In this regard, ATRA is of particular interest due to its strong antitumor activity and convenience for use. The use of ATRA plus chemotherapeutic agents for the treatment of solid tumors has been previously investigated (38); however, ATRA was shown to be extremely toxic hampering its use in clinical practice (39). This issue prompted us to focus on the activity of ATRA at a non-toxic concentration in our study.

At the low concentrations tested in this study, ATRA showed no significant cytotoxic effect on the HepG_2_ cell line, but effectively inhibited SMMC-7721 cell growth in a dose-dependent manner (Fig. 1B). These results are in agreement with prior data showing that HepG_2_ cells were more resistant to ATRA treatment, and were only susceptible to an extremely high concentration of ATRA (20). Data from the MTT assay showed that sorafenib alone had a significant effect on cell viability only at relatively high concentrations in both HCC cell lines (Fig. 1A). Of note, when combined with ATRA at a non-toxic concentration, there was substantial enhancement of the effect of sorafenib in inducing both cell growth inhibition and apoptosis (Figs. 1 and 2). The concentration of sorafenib used in the combination study with ATRA was approximately the dose that is used in most *in vitro* studies (2.5–10 μM) (3,40,41), and within peak plasma levels of sorafenib (5–10 μg/ml) that can be achieved by oral administration (8,42). The use of these doses led to a moderate inhibition of cell growth and induction of apoptosis by sorafenib, and allowed us to reveal clinically relevant modulation of the effects of sorafenib in combination with ATRA.

We further demonstrated that the increased apoptosis induced by sorafenib plus ATRA was GJIC-dependent, since pre-treatment with GJIC inhibitors dramatically blocked the apoptosis induced by sorafenib plus ATRA (Fig. 3). Subsequent experiments revealed that treatment with sorafenib and ATRA alone led to a moderately positive effect on gap junction function, while combined treatment resulted in a strong synergistic enhancement in GJIC activity (Fig. 4), further strengthening this conclusion. More importantly, the dose at which sorafenib or ATRA positively modulated GJIC, was minimally toxic for both cell lines. Low toxicity but effective concentrations of either drug in clinical practice are needed, and the effect of cell toxicity on GJIC must be excluded.

The drug-induced gap junction stimulation in tumor cells is important in cancer therapy, since aberrant GJIC is widely regarded to correlate with tumorigenic phenotypes (28,29). However, changes in the status of gap junction function with tumorigenesis are complex and heterogeneous. In some cases, such as ovarian adenocarcinomas and cervical cancer, GJIC is dramatically reduced or essentially absent (43,44). In other cases, GJIC is maintained or only modestly reduced. For example, in the liver, GJIC involves at least three different Cxs, Cx32, Cx26 and Cx43, depending on the cell type or cell position in the lobule (45). In hepatoma cell lines, Cx26 is normally not detected, while Cx43 is upregulated and Cx32 is upregulated or downregulated depending on the type of tumor (31,37,46). Consistent with these previous reports, only Cx32 and Cx43 proteins were detectable in both HCC cell lines (Fig. 5A). HepG_2_ and SMMC-7721 cells are gap junctional communication-competent cells, as evidenced by the dye transfer assay to verify the presence of functional GJIC (Fig. 3A). Thus, in this situation, one would expect GJIC-mediated cell growth control to play a role.

GJIC-mediated cell sensitivity to anticancer therapy has been demonstrated in many systems (25–27). The mechanism involves the transport of anticancer agents or their active metabolites to adjacent cells through gap junctions, thereby targeting a greater proportion of the cell population. Most relevant to our study, upregulation of GJIC by cyclic-AMP and ATRA has been shown to act synergistically with a variety of chemotherapeutic agents to cause cell death (47). Some toxic metabolites of prodrugs (e.g., ganciclovir triphosphate and 5-fluorouracil) can pass between cells through gap junctions (48,49). Since the bystander toxic effect depends on the gap junction level, and the functionality of this pathway in human cancers probably provides an important determinant of the clinical response to anticancer agents, prevention of GJIC downregulation and restoration of GJIC in tumor cells could be a rational chemopreventive approach. In our case, although GJIC was moderately enhanced by either sorafenib or ATRA alone, the role of GJIC in cell growth control was not triggered and the growth-inhibitory effect by either agent was slight. However, when the two agents were concurrently administered, there was substantial enhancement of the toxic effect by the two agents at which condition the GJIC was markedly enhanced. We hypothesized that the toxic signals generated in one cell can enter another via GJIC and thus enhance the likelihood of cell death in a cell that might not otherwise be affected by either agent alone, and in turn, increased drug sensitivity may be generated in a positive feedback mechanism.

In addition to GJIC-mediated toxic effects, gap junctions also play an important role in the control of cell growth and differentiation. Evidence in support of this indicates that the disruption of GJIC and abnormal expression of Cxs have been found in transformed and cancer cell lines (28,29), while GJIC recovery in these cells results in growth normalization and suppression of tumorigenicity (28,50). Moreover, studies have shown that the antitumor effects of various antineoplastic agents, including ATRA, are associated with restoration of GJIC function and Cx expression in a number of solid tumors (21,22,37). These results suggest that Cxs may be defined as tumor suppressors and that restoration of GJIC may be a unique antitumor therapeutic strategy. Herein, the ability of sorafenib at a low concentration to enhance GJIC is important, since this study is the first to explore the upregulation of gap junction function in HCC cell lines by sorafenib, which will add a new component to its mechanistic frame.

In exploring the mechanisms by which GJIC was enhanced, we found a slight increase in expression of Cx43, but not Cx32, by a transcriptional mechanism following sorafenib or ATRA treatment alone in HepG_2_ cells. SMMC-7721 is a poor differentiated primary human HCC cell line derived from an elderly Chinese patient. The cells possess high capacity for tumorigenicity and low capacity for metastasis (51), for which the GJIC characteristic has not been entirely ruled out. In contrast to the HepG_2_ cell line, a significant increase in both protein and mRNA levels of Cx32, but not Cx43, was observed after 48 h of each drug treatment in SMMC-7721 cells. Hence, the increase in Cx43 expression in HepG_2_ cells, and Cx32 expression in SMMC-7721 cells, could be interpreted as one main cell event responsible for gap junction upregulation of the targeted cells. The reasons responsible for these cell-type discrepancies are unknown, although they may be relative to cell type-specific transcriptional regulators. Since differential expression of Cxs in a variety of tissues is generally believed to reflect cell-specific regulation of junctional coupling and functional demands for GJIC in different cell types, it is possible that a particular Cx may function as a tumor modulating protein in one or several specific cell types but not in others. Although the patterns of action were not entirely identical, a common feature of the cell response to treatment was that sorafenib and ATRA acted synergistically, under each condition, to induce a more significant increase in both protein and mRNA levels of its structural Cx. The upregulation of Cx expression is also related to the increase in the number of Cx channels on the cell membrane as evidenced by the immunofluorescence assay (Fig. 6). These data strongly suggest that enhanced GJIC was correlated with upregulation of Cx relocalization and protein amount by a transcriptional mechanism. In future studies, the observed sorafenib effects should be confirmed in other cancer cell lines with different Cx expression profiles and GJIC levels.

Taken together, the data, presented here for the first time, suggest that sorafenib and ATRA act synergistically to enhance inhibition of cell growth and induction of apoptosis via the upregulation of Cx expression and relocalization resulting in the enhancement of GJIC in HCC cell lines. Thus, the combination treatment represents a future therapeutic option for the treatment of HCC.
